# Hereditary Gastric Cancer: Single-Gene or Multigene Panel Testing? A Mono-Institutional Experience

**DOI:** 10.3390/genes14051077

**Published:** 2023-05-13

**Authors:** Mariarosaria Calvello, Monica Marabelli, Sara Gandini, Elena Marino, Loris Bernard, Matteo Dal Molin, Giulia Di Cola, Cristina Zanzottera, Giovanni Corso, Nicola Fazio, Lorenzo Gervaso, Uberto Fumagalli Romario, Massimo Barberis, Aliana Guerrieri-Gonzaga, Lucio Bertario, Davide Serrano, Bernardo Bonanni

**Affiliations:** 1 Division of Cancer Prevention and Genetics, IEO, European Institute of Oncology IRCCS, 20141 Milan, Italy; 2 Department of Experimental Oncology, IEO, European Institute of Oncology IRCCS, 20141 Milan, Italy; 3 Clinic Unit of Oncogenomics, IEO, European Institute of Oncology IRCCS, 20141 Milan, Italy; 4 Division of Breast Surgery, IEO, European Institute of Oncology IRCCS, 20141 Milan, Italy; 5 Department of Oncology and Hemato-Oncology, University of Milan, 20122 Milan, Italy; 6 European Cancer Prevention Organization (ECP), 20122 Milan, Italy; 7 Division of Gastrointestinal Medical Oncology and Neuroendocrine Tumors, IEO, European Institute of Oncology IRCCS, 20141 Milan, Italy; 8 Molecular Medicine Program, University of Pavia, 27100 Pavia, Italy; 9 Digestive Surgery, IEO, European Institute of Oncology IRCCS, 20141 Milan, Italy

**Keywords:** gastric cancer, multigene panel testing, *BRCA1*, *BRCA2*, *CDH1*, *MSH2*, hereditary diffuse gastric cancer syndrome, homologous recombination DNA repair genes

## Abstract

Gastric cancer (GC) has long been a ‘Cinderella’ among hereditary cancers. Until recently, single-gene testing (SGT) was the only approach to identify high-risk individuals. With the spread of multigene panel testing (MGPT), a debate arose on the involvement of other genes, particularly those pertaining to homologous recombination (HR) repair. We report our mono-institutional experience in genetic counseling and SGT for 54 GC patients, with the detection of nine pathogenic variants (PVs) (9/54:16.7%). Seven out of fifty (14%) patients who underwent SGT for unknown mutations were carriers of a PV in *CDH1* (n = 3), *BRCA2* (n = 2), *BRCA1* (n = 1), and *MSH2* (n = 1), while one patient (2%) carried two variants of unknown significance (VUSs). *CDH1* and *MSH2* emerged as genes involved in early-onset diffuse and later-onset intestinal GCs, respectively. We additionally conducted MGPT on 37 patients, identifying five PVs (13.5%), including three (3/5:60%) in an HR gene (*BRCA2*, *ATM*, *RAD51D*) and at least one VUS in 13 patients (35.1%). Comparing PV carriers and non-carriers, we observed a statistically significant difference in PVs between patients with and without family history of GC (*p*-value: 0.045) or Lynch-related tumors (*p*-value: 0.036). Genetic counseling remains central to GC risk assessment. MGPT appeared advantageous in patients with unspecific phenotypes, although it led to challenging results.

## 1. Introduction

Gastric cancer (GC) still represents a significant health problem worldwide. Despite a decrease in incidence and mortality, mainly due to the diffusion of *Helicobacter pylori* (*H. pylori*) eradication therapies and the improvement of hygienic and economic conditions, GC remains the fifth most common cancer and the fourth leading cause of cancer-related death [1]. Additionally, GC ranks among cancers with the worst prognosis, principally linked to diagnostic delay. Thus, the best strategy to prevent GC includes the reduction of exposure to known risk factors (*H. pylori* infection, alcohol, smoking, obesity, radiation, and others) and early detection [2].

Positive family history (FH) and genetic defects associated with hereditary cancer predisposition syndromes also enhance GC risk, accounting for about, respectively, 10% and 1–3% of GCs overall [3]. The three main cancer predisposition syndromes responsible for an increased GC risk are hereditary diffuse gastric cancer (HDGC), gastric adenocarcinoma and proximal polyposis of the stomach (GAPPS), and familial intestinal gastric cancer (FIGC) [4].

HDGC is an autosomal dominant disorder, mainly due to pathogenic variants (PVs) in *CDH1* and, more rarely, *CTNNA1* genes, and typically associated with diffuse gastric cancer (DGC) and lobular breast cancer. Germline genetic testing of *CDH1* and, recently, *CTNNA1* is recommended when an individual fulfills the clinical criteria according to the updated guidelines of the International Gastric Cancer Linkage Consortium (IGCLC) [5].

GAPPS is another autosomal dominant GC hereditary predisposition syndrome. Only germline single-nucleotide variants located in the promoter 1B of the *APC* gene are responsible for GAPPS, currently recognized as a gastric-restricted variant of familial adenomatous polyposis (FAP). GAPPS-associated GCs arise characteristically in the context of a proximal fundic gland polyposis [6]. Updated clinical criteria in 2018 overcame the 2012 criteria, initially established at the time of the first GAPPS description [7], and included the presence, in the index case, of at least 100 polyps—predominantly fundic gland polyps—of the proximal stomach with colorectal and duodenal sparing and an autosomal pattern of inheritance, with the exclusion of other genetic conditions associated with gastric polyposis and the use of proton pump inhibitors [8].

Unlike HDGC and GAPPS, FIGC, the third major cancer predisposition syndrome involving GC, remains genetically undetermined. In 1999, the IGCLC first established the clinical diagnostic criteria for FIGC [9]. Lately, simplified criteria have been proposed for use in the diagnosis of FIGC patients [10,11].

In addition to these three classic GC predisposition syndromes, other conditions appear to be involved in conferring GC risk (i.e., FAP, Lynch syndrome, Peutz–Jeghers syndrome, juvenile polyposis, Li–Fraumeni syndrome, and *MUTYH*-associated polyposis). There is an active debate on the possible involvement of candidate genes such as *MSH2* in FIGC; *RAD51C* and others in HDGC; and *BRCA1*, *BRCA2*, *ATM*, and *PALB2* in both HDGC and FIGC [11].

Identifying carriers of germline PVs in GC cancer predisposition genes may prompt target preventive strategies (surveillance or, when applicable, risk-reducing surgery) and represents an opportunity to carry out a cascade screening, leading to early detection, an indirect improvement in the prognosis of cancer, and a decrease in mortality. Of relevance, a recent study showed that GC risk was significantly higher in carriers of a PV in a homologous recombination (HR) DNA repair gene who had an *H. pylori* infection than in subjects with one factor alone [12].

Hence, evidence of the possible involvement of several different genes in GC risk recently advised the employment of multigene panel testing (MGPT), especially for research purposes, as it is more advantageous compared to the traditional single-gene testing (SGT) approach (i.e., the analysis of one or more specific genes in patients fulfilling the established selection criteria for genetic testing) [13]. However, the use of MGPT, especially in unselected GC patients, is not free of knotty results such as the unexpected detection of PVs in genes not strictly associated with the phenotype (secondary findings) or, more frequently, the high rate of variants of unknown significance (VUSs). These results can lack clinical utility and remarkably complicate patient/family management [5,14].

Here, we report our mono-institutional experience in genetic GC risk assessment at the European Institute of Oncology (IEO), a specialist third-level center. In this study, we evaluated the performance of genetic counseling and SGT to identify high-risk individuals among GC patients. We extended the analysis to additional genes to verify whether MGPT can provide clinically actionable results, leading to the identification of more high-risk GC patients. More specifically, we explored the possible involvement of other genes, such as HR DNA repair genes, in GC predisposition. Lastly, we compared carriers and non-carriers to outline clinical features of GC patients that can point clinicians towards the most successful approach for genetic testing.

## 2. Materials and Methods

### 2.1. Patients’ Selection

**Figure 1:**
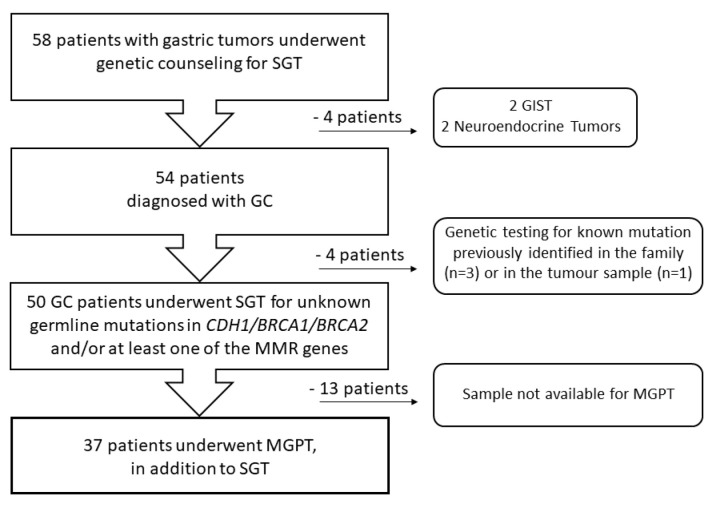
Flow diagram of participant selection. GC—gastric cancer, GIST—gastrointestinal stromal tumor, MGPT—multigene panel testing, MMR—mismatch repair, SGT—single gene testing.

Fifty-eight patients with a diagnosis of a gastric tumor and without proximal gastric polyposis were consecutively referred for genetic counseling to the Division of Cancer Prevention and Genetics at IEO between 2002 and 2022. They underwent germline SGT for one or more genes (including *BRCA1*, *BRCA2*, *CDH1*, *MLH1*, *MSH2*, *MSH6*, *PMS2*, and/or *EPCAM*) according to their personal history (PH) and/or FH. As shown in Figure 1, in this study, we retrospectively analyzed 54 patients with gastric adenocarcinoma and excluded four patients with a diagnosis of gastric neuroendocrine tumor (n = 2) or gastrointestinal stromal tumor (GIST; n = 2).

A detailed FH (information from at least three generations of relatives) was obtained. The clinical and genetic data were collected and stored in a dedicated institutional database. Additional data were collected using medical records and pathology reports. GCs were distinguished as intestinal gastric cancer (IGC), DGC, or mixed type, according to the Lauren histological classification [15], and in early (stage 0, I, IIA, and IIB) or late stage (stage III, IVA, and IVB), as per the American Joint Committee on Cancer (AJCC) prognostic stage groups [16].

We revised genetic testing criteria for all patients following the NCCN Version 1.2020 for *BRCA1* and *BRCA2* genes [17], the HDGC 2020 criteria for the *CDH1* gene [5], and PREMM5 Model [18] score or tumor testing (i.e., microsatellite instability (MSI) evaluation and/or immunohistochemistry for the mismatch repair (MMR) proteins) results, when available, for one or more MMR (including *MLH1*, *MSH2*, *MSH6*, and *PMS2*) and *EPCAM* genes. We classified as FIGC all patients with isolated IGC < 70 years old or families with ≥2 GC cases, one confirmed IGC, according to the definition of FIGC recently used by Garcia-Pelaez et al. [11].

In addition to SGT, 37 out of 54 patients with an available peripheral blood sample stored in our biobank underwent MGPT including 29 cancer-related genes.

The study was conducted in accordance with the Declaration of Helsinki and approved by the Institutional Review Board of IEO (protocol code UID 4034). All participants provided an informed consent.

### 2.2. DNA Extraction and Quantification

Blood samples were collected from all 54 GC patients involved in the study using standard procedures. gDNA extraction was carried out starting from 400 μL of whole peripheral blood using a MagCore Super Automated Nucleic Acid Extractor (Diatech Pharmacogenetics srl, Jesi, Italy), following the manufacturer’s protocol (Ver.20210503.01). First DNA quantification was provided by the MagCore Extractor. For NGS library preparation, DNA was further quantitated using the Qubit dsDNA HS Assay kit with the Qubit 3.0 Fluorometer (Life Technologies, Carlsbad, CA, USA), following the protocol provided by the manufacturer.

### 2.3. Single Gene Testing

All 54 patients were referred to SGT for specific genes, according to their PH and/or FH. SGT for unknown germline mutations in one or more genes was carried out by standard PCR and Sanger sequencing/multiplex ligation-dependent probe amplification in 50 patients: 43 in *CDH1*, nine in *BRCA1*/*BRCA2*, and 12 in at least one of the *MMR*/*EPCAM* genes. Three patients underwent germline SGT for known familial PVs (one in *BRCA1*, one in *BRCA2*, and one in the *MSH2* gene). The last patient underwent germline SGT because of the identification of a PV in *CDH1* in the tumor sample, to verify its possible germinal origin.

### 2.4. MGPT Using Hereditary Cancer Solution by Sophia Genetics

Thirty-seven GC patients with available blood samples underwent MGPT. This approach included both patients with negative SGT results and patients already proven to be PV/VUS carriers by SGT, to explore the possible involvement of other GC candidate genes. We performed MGPT using the Hereditary Cancer Solution CE-IVD by Sophia Genetics, according to protocols provided by the manufacturer (version PM_T1_5.1.5_r2en July 2017). This custom panel covers the coding regions and 25 bp of exon flanking non-coding DNA of the 29 most clinically relevant genes associated with hereditary cancer syndromes (Table S1). Library preparation was optimized for 200 ng of total gDNA (as quantified with a Qubit 3.0 Fluorometer, Life Technologies), using an enrichment protocol for the simultaneous sequencing of 29 genes. Results were retrieved and analyzed with SOPHIA DDM. Libraries were quantified using the 4200 TapeStation (Agilent Technologies, Santa Clara, CA, USA) and Qubit 3.0 Fluorometer (Life Technologies) and diluted to 4 nM. Following denaturation, a 10 pM library dilution was loaded with 3% PhiX control (Illumina, San Diego, CA, USA). Sequencing was performed with the Illumina MiSeq system, using the MiSeq V2 Standard reagent Kit 2 × 250 cycles.

### 2.5. Variant Classification

The identified genetic variants were divided into five classes according to the International Agency for Research on Cancer (IARC) recommendations [19]. Variant pathogenicity was assessed using the ClinVar database (https://www.ncbi.nlm.nih.gov/clinvar/ (accessed on 7 April 2023)), the Leiden Open Variation Database (LOVD) (https://www.lovd.nl/ (accessed on 7 April 2023)), the BRCA Exchange (https://brcaexchange.org/ (accessed on 7 April 2023)) for *BRCA1* and *BRCA2* genes, and following the ACMG guidelines [20]. In this study, both pathogenic (C5) and likely pathogenic (C4) variants were defined as PVs.

### 2.6. Statistical Analysis

The absolute and relative frequencies of the 37 patients who underwent MGPT, both carriers and non-carriers, were presented by demographic, clinical, and histopathological features described as categorical variables. Given the low frequency of carriers, Fisher’s exact test was used to assess the associations of carrier status with those characteristics. The Wilcoxon rank test was used to compare continuous variables. Two-sided *p* < 0.05 was considered statistically significant. The statistical analyses were performed with the Statistical Analysis System Version 9.2 (SAS Institute, Cary, CA, USA).

## 3. Results

### 3.1. Description of GC Patients

Table 1 summarizes the characteristics of the patients involved in this study. Out of 54 GC patients, 37 (68.5%) were female and 17 (31.5%) were male. The mean age at diagnosis was 44.4 years (range 23–71). According to the Lauren histological classification, 8 (14.8%) GCs were intestinal, 33 (61.1%) were diffuse, and 8 (14.8%) were of mixed type. The Lauren histological classification was not available for five (9.3%) patients. The majority of GCs were distally located (i.e., excluding cardia) (42/54 = 77.8%). Only seven GCs arose in cardia (7/54 = 12.9%). The anatomic location was unknown in five cases (9.3%). Data about *H. pylori* infection was available for 20 patients (37%). Eleven patients had a PH positive for an *H. pylori* infection. Twenty-seven (50%) GCs were diagnosed at an early stage (0–II stage) and 24 (44.4%) at a late stage (III–IV stage), according to AJCC staging at diagnosis. The staging was unknown or impossible to characterize for three GCs (5.6%). Fourteen (14/54 = 25.9%) patients developed extragastric cancers. Seven (13%) patients developed at least one breast cancer (BC): four patients were diagnosed with invasive ductal carcinoma, two with a ductal metachronous bilateral BC, and one with a lobular metachronous bilateral BC. Seven patients were affected by other primary tumors: three by colorectal adenocarcinomas (CRCs), one by ovarian cancer, and three by cancers in different sites. Nine out of fourteen patients with extragastric cancers, meaning 16.7% of all patients, developed additional primary tumors. FH was available for all patients except for one. Forty-eight (48/54 = 88.9%) patients reported a positive FH. Namely, FH was positive for GC in twenty-seven patients, specifically for DGC in four, IGC in four, and not otherwise specified GC in nineteen patients. Twenty-five patients reported FH-positive for tumors frequently observed in the *BRCA*-associated hereditary breast and ovarian cancer (HBOC) syndrome (i.e., breast, ovarian, and prostate cancer), eighteen patients for tumors associated with Lynch syndrome (LS) (i.e., CRC, endometrial cancer, small intestine, urinary tract/bladder/kidney, bile ducts, brain, and sebaceous gland skin tumors), and five patients for pancreatic cancer, belonging both to HBOC- and LS-related tumors.

A detailed description of all clinical features as well as personal and family history of the 54 GC patients has been reported in Table S2.

### 3.2. SGT Results

Overall, SGT led to positive results (i.e., to the detection of a PV) in nine patients (9/54 = 16.7%). As reported in Table 2, seven out of the fifty (7/50 = 14%) patients who underwent SGT for unknown mutations in one or more genes were carriers of a PV, namely in *CDH1* (n = 3), *BRCA2* (n = 2), *BRCA1* (n = 1), and *MSH2* (n = 1). Two out of the four patients who were addressed to SGT for known mutations were carriers of PVs, specifically in the *MSH2* and *BRCA2* genes. One out of three *CDH1* carriers met *BRCA1*/*BRCA2* criteria in addition to HDGC 2020 criteria. The *MSH2* carrier fulfilled only the proposed FIGC criteria [11], and for this patient, the PREMM5 score was ≥50%. Of note, this patient also developed an MSI-High CRC with a defective expression of the *MSH2* and *MSH6* proteins. Out of 43 patients who underwent SGT to investigate the presence of unknown mutations of the *CDH1* gene, 32 (32/43 = 74.4%) fulfilled HDGC 2020 criteria, and 3 (3/32 = 9.4%) were carriers of a PV in *CDH1*. SGT for *CDH1* did not detect PVs in patients who did not meet HDGC 2020 criteria. Three out of seven (3/7 = 42.9%) patients who fulfilled *BRCA1* and *BRCA2* genetic testing criteria and underwent SGT for the search of unknown mutations were carriers of one PV in *BRCA1* and two PVs in *BRCA2*, respectively. SGT led to uncertain results only in one case (1/50 = 2%) found to be a carrier of two VUSs in *BRCA2*.

### 3.3. MGPT Results

MGPT was offered to 37 out of the 54 GC patients (68.5%) (Table 2). This additional germline test by MGPT drove positive results in five patients (5/37 = 13.5%). Three of these PVs were previously identified by the SGT as well (one in *BRCA2*, one in *MSH2*, and one in *CDH1*). These PV carriers met the criteria for *BRCA1*/*BRCA2* genetic testing, the HDGC 2020 criteria, and the FIGC criteria, respectively. Two additional PVs were detected by MGPT in the *ATM* and *RAD51D* genes.

MGPT led to uncertain results (detection of at least one VUS) in 13 patients (13/37 = 35.1%) and, overall, a challenging result (VUS and PV in possible GC candidate genes) in 15 patients (15/37 = 40.5%).

Overall, 11 PVs have been identified by SGT and/or MGPT (listed in Table 3).

Table S3 shows all genetic testing results from both SGT and MGPT, including details on both PVs and VUSs.

Table 4 shows the demographic, clinical, and histopathological features of the 37 GC patients, PV carriers and non-carriers, who underwent MGPT. Three out of eight (37.5%) patients with a diagnosis of multiple primary tumors were carriers of a PV. Of note, a significantly higher detection of PVs was observed in patients with a positive FH of GC (5/18 = 27.8%) compared to patients without FH of GC (0/18 = 0%) (*p*-value: 0.045). Similarly, we found a significant statistical difference between patients with a positive FH of LS-related tumors (4/12 = 33.3%) and without FH of LS-related tumors (1/24 = 4.2%) (*p*-value: 0.036). Regarding the histological classification, no statistical differences were observed between PV detection in different groups (2/24 = 8.3% DGC, 2/3 = 66.7% IGC, 0/5 = 0% mixed type GC, 1/5 = 20% not otherwise specified GC; *p*-value: 0.075). However, the detection of PV by MGPT appears to be significantly higher in patients with IGC (2/3 = 66.7%) than in those with DGC (2/24 = 8.3%) (*p*-value:0.0485).

### 3.4. Age Distribution and GC Histological Classification in PV Carriers

**Figure 2:**
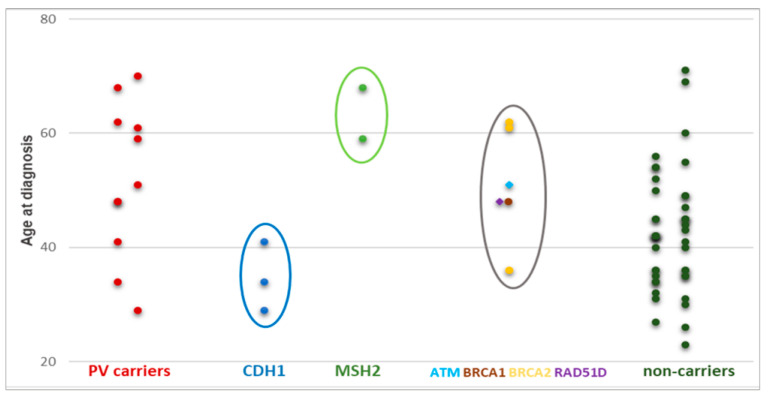
Distribution of age at GC diagnosis in carriers and non-carriers. Circles highlight the different distribution of age at GC diagnosis between PV carriers in *CDH1* (blue), *MSH2* (green), and HR DNA repair genes (grey).

Figure 2 shows the distribution of age at GC diagnosis of the 11 PV carriers (identified by SGT and/or MGPT) and of the non-carriers. Of note, *CDH1* and *MSH2* PV carriers appeared to have an earlier and later onset of GC, respectively. On the other hand, the distribution of age at diagnosis appeared to be variable in carriers of a PV in GC candidate genes (belonging to the HR pathway), such as *ATM*, *BRCA1*, *BRCA2*, and *RAD51D*. *CDH1* PV carriers developed only DGCs (3/3 = 100%), while *MSH2* PV carriers only IGCs (2/2 = 100%). Conversely, GC patients who carry a PV in HR DNA repair genes presented DGC, IGC, and other histotypes.

## 4. Discussion

In this study, we reported our mono-institutional experience in genetic counseling and testing for GC patients. First, we retrospectively analyzed 54 patients diagnosed with GC who underwent SGT according to their personal or family history. Overall, SGT detected a PV in cancer predisposition genes in about 17% of our GC patients, demonstrating that accurate genetic risk assessment through pre-counseling is an optimal approach to identify high-risk individuals. SGT for unknown mutations, conducted in about 93% of patients, also showed a high detection of PVs and, interestingly, a low rate (about 2%) of VUSs. These results could be mainly explained by the stringent selection criteria applied for genetic testing. In this regard, 9.4% of patients who fulfilled HDGC 2020 criteria were carriers of a PV in *CDH1*.

However, the SGT approach has inevitably resulted in some limitations and discrepancies with the literature. In contrast with the reported data [1], 68.5% of our GC patients were female. Together with the low mean age at GC diagnosis (44.4 years), the prevalence of DGCs (61.1%), the high percentage of cases with multiple primary tumors (25.9%), and positive FH (88.9%), this reflects the selection criteria used for genetic testing. In fact, we observed that, overall, 29.6% (16/54) of patients met the *BRCA1/BRCA2* genetic testing criteria, thus explaining the high percentage of female GC patients. Moreover, most of our GC patients (~80%) were referred for *CDH1* SGT testing following the HDGC 2020 criteria, consequently justifying the high frequency of DGC in our study. According to previously reported anatomical subsites, the majority (79.6%) of GCs arose in the distal region of the stomach. Distal GCs are classically defined non-cardia and are generally associated with *H. pylori* infection and other risk factors [1]. However, in our series, information on *H. pylori* was available only for 37%, and infection was found in 11 patients (11/20 = 55%).

For many years, the SGT has been the only or the most commonly used approach for genetic testing. Moreover, for a long time, hereditary GCs remained in a “Cinderella” status, neglected and underinvestigated, being very few the cancer predisposition syndromes recognized as associated with GCs (FIGC, LS, GAPPS, and HDGC). The debate concerning a possible role of other genes in hereditary GC began only in the last few years, with the spread of MGPT in the research setting and the achievement of some evidence concerning a possible association with GC risk, especially in *BRCA2, PALB2, ATM*, and other DNA repair gene PV carriers [11,12,21,22].

In light of these latest data, we explored the involvement of other genes and the suitability of the clinical use of the MGPT approach in differently selected GC patients. The MGPT evidenced, in addition to the PVs previously identified by SGT, two further PVs in the *ATM* and *RAD51D* genes. Contrary to *ATM*, previously reported as a possible candidate gene for both HDGC and FIGC, *RAD51D* was detected in only a few cases of GC patients [23]. Interestingly, the *ATM* PV carrier presented personal and family history strongly consistent with the typical clinical picture associated with this gene, being the patient diagnosed with pancreatic cancer in addition to gastric cancer and having a daughter with early-onset breast cancer. On the contrary, the personal and family history of the *RAD51D* carrier was negative for ovarian cancer, which is typically associated with this gene, and for breast cancer, except for one second-degree relative. Indeed, this patient presented the typical clinical picture of hereditary gastric cancer, suggesting *RAD51D* as a possible candidate gene for this condition, as previously proposed for *RAD51C* [11].

Recently, an increased risk of GC was reported for carriers of a PV in an HR DNA repair gene (particularly *ATM*, *BRCA1*, *BRCA2*, and *PALB2*) with *H. pylori* infection [12], leading the authors to consider evaluation and eradication of *H. pylori* as strongly recommended in carriers of a PV in an HR DNA repair gene. Although this evidence reveals the role of HR DNA repair genes in GC predisposition, clinical management of carriers of a PV in moderate-penetrance genes (such as *ATM* and *RAD51D*) is still puzzling [10,12]. In addition to these complex findings, MGPT can result in a high detection rate of VUSs. In this study, we detected at least one VUS in about 35.1% of patients, a lower rate than previously reported [24], probably due to the minor number of genes we included in the MGPT.

Regarding the involvement of HR DNA repair genes, here we report a detection rate of 8.1%, similar to that previously described by Uson et al. (3/34 GC patients: 8.8%) [24], but slightly lower than that by Zhang et al., who recently reported a 10% of detection rate of PVs in HR DNA repair genes in GC patients [25]. Lu et al. found that, after ovarian cancer, GCs are the cancers with the highest percentage of germline truncating variants in genes of the Fanconi anemia pathway [26]. All these results led some authors to research new tools and assays capable of investigating HR deficiency as a signature for prognostic scores or response to treatment in GCs [27]. In the context of metastatic GC, poly (ADP-ribose) polymerase inhibitors (PARPi) have been studied in an unselected population with limited results in terms of overall response rate in a phase I trial [28]. Currently, several other clinical trials are investigating the role of PARPi in GC in combination with immunotherapy or chemotherapy (NCT04276376, NCT03829345, NCT03840967).

Comparing carriers with non-carriers according to MGPT results, we concluded that this approach could be more helpful in GC patients with a positive FH of LS-related cancers and GC than in those with a positive FH of HBOC-related cancers. Unexpectedly, we did not detect a significantly higher percentage of PVs in GC patients considering all the histological subtypes, probably due to the size of our series. However, we found a statistically significant difference between the detection of PVs in IGCs versus DGCs, suggesting that the MGPT approach may be more advantageous in selected IGCs than in selected DGCs. Interestingly, by comparison with the literature, we also observed a difference between the detection of *CDH1* PVs by SGT in our selected GC patients and by MGPT in previously reported unselected patients [23,26]. Therefore, *CDH1* genetic testing should preferably be recommended according to specific selection criteria. Remarkably, it is well-known that unexpected PVs in *CDH1* represent clinically challenging results of MGPT carried out in unselected patients [14].

Concerning the clinical features of GCs developed by PV carriers, we noted an earlier age at diagnosis in *CDH1* PV carriers than in *MSH2* PV carriers. According to previous data, germline *CDH1* PVs are generally responsible for early GC onset, with an average age at diagnosis of 38 years [29]. On the other hand, as previously reported [30], germline *MSH2* PVs can be associated with a later GC onset, acting as moderate penetrance risk factors for GC. A variable age distribution for GC diagnosis characterized patients with PVs in HR DNA repair genes, such as *BRCA1*, *BRCA2*, *RAD51D*, and *ATM*. Regarding histological features, *CDH1* PVs were detected only in patients with DGCs and *MSH2* PVs were detected only in patients with IGCs. As previously reported [10], we found PVs in the HR DNA repair genes of GC patients with different histotypes.

The main limitation of this retrospective observational study is the small sample size. Due to its exploratory nature, we did not adjust for multiple testing when generating *p*-values in our analysis. Thus, given the low statistical power, results should be considered suggestive and not conclusive. Moreover, regrettably, GC patients fulfilling HDGC 2020 criteria did not undergo *CTNNA1* SGT, although it is recommended according to recent guidelines [5].

Following recent evidence that HR DNA repair genes are involved in GC predisposition, further studies are needed to investigate them as possible markers of prognosis or as a response to specific target therapies.

## 5. Conclusions

Taken together, our results led to the following conclusions: (1) SGT can still represent a proper approach for the detection of germline PVs in patients referred to genetic counseling according to specific selection criteria. (2) SGT appears to be the best approach to detect *CDH1* PVs in patients fulfilling HDGC 2020 selection criteria. (3) In addition to the high PV rate, the SGT approach had also the great advantage of a low detection rate of VUSs or other challenging results (e.g., secondary findings). (4) MGPT could be a proper approach for patients with a diagnosis of IGC, positive FH of GC or LS-related cancers, as these phenotypes overlap different cancer predisposition syndromes, contrary to DGC typically associated with HDGC. (5) MGPT led to uncertain/complex results in about 41% of GC patients, including PV identification in possible candidate genes not yet clearly associated with hereditary GCs. (6) MGPT identified a PV in HR DNA repair genes in about 8% of our GC patients. (7) Further studies are needed to investigate the involvement of HR DNA repair genes in GC risk, prognosis, and treatment.

## Figures and Tables

**Table 1:** Characteristics of the 54 patients with gastric adenocarcinoma.

Variables	N°	%
**Sex**		
Male	17	31.5%
Female	37	68.5%
**Age at diagnosis**		
<50	40	74.1%
≥50	14	25.9%
**Histology according to Lauren**		
Diffuse	33	61.1%
Intestinal	8	14.8%
Mixed	8	14.8%
Unknown	5	9.3%
**Anatomic location**		
Cardia	7	12.9%
Non-cardia	42	77.8%
Unknown	5	9.3%
***H. pylori*** **infection**		
Yes	11	20.4%
No	9	16.7%
Unknown	34	62.9%
**AJCC Staging at diagnosis**		
Early Stage	27	50.0%
Late Stage	24	44.4%
Unknown	3	5.6%
**Other tumors**		
Yes	14	25.9%
No	40	74.1%
**Family history of GC**		
Yes	27	50.0%
No	26	48.1%
Unknown	1	1.9%

AJCC American Joint Committee on Cancer, *H. pylori* Helicobacter pylori.

**Table 2:** Genetic testing results from both SGT and MGPT.

	N°	%
SGT *		
Yes	50	92.6%
Genes analyzed by SGT *		
*CDH1*	43	86.0%
*BRCA1*, *BRCA2*	9	18.0%
*MLH1*, *PMS2*	10	20.0%
*MSH2*, *MSH6*, EPCAM **	11	22.0%
SGT * results		
positive	7	14.0%
*CDH1*	3	
*BRCA2*	2	
*BRCA1*	1	
*MSH2*	1	
uncertain	1	2.0%
uninformative	42	84.0%
**MGPT**		
Yes	37	68.5%
No	17	31.5%
**MGPT results**		
positive	5	13.5%
*CDH1*	1	
*BRCA2*	1	
*MSH2*	1	
*ATM*	1	
*RAD51D*	1	
uncertain	13	35.1%
uninformative	19	51.4%

* search for unknown mutations only. ** search for large rearrangements only.

**Table 3:** List of pathogenic variants (PVs) identified by SGT and/or MGPT.

ID	Gene	cDNA Variant *	Protein Variant	Classification	Gene Testing Approach
**2**	*MSH2*	c.1216C>T	p.Arg406Ter	pathogenic	SGT
**7**	*BRCA2*	c.8633-1G>A	p.?	likely pathogenic	SGT
**13**	*RAD51D*	c.1A>G	p.Met1Val	likely pathogenic	MGPT
**23**	*MSH2*	c.339_340del	p.Asn115Ter	pathogenic	SGT/MGPT
**24**	*BRCA1*	c.5278-2A>T	p.?	likely pathogenic	SGT
**38**	*CDH1*	c.1792C>T	p.Arg598Ter	pathogenic	SGT
**40**	*CDH1*	c.833-476_1138-464del	p.Gly278ValfsTer7	pathogenic	SGT
**42**	*ATM*	c.2413C>T	p.Arg805Ter	pathogenic	MGPT
**48**	*BRCA2*	c.67+1G>A	p.?	likely pathogenic	SGT
**49**	*BRCA2*	c.67+1G>A	p.?	likely pathogenic	SGT/MGPT
**53**	*CDH1*	c.2416G>T	p.Glu806Ter	likely pathogenic	SGT/MGPT

* Reference sequences (Human Feb. 2009—GRCh37/hg19 Assembly): *ATM* NM_000051.4; *BRCA1* NM_007294.4; *BRCA2* NM_000059.4; *CDH1* NM_004360.5; *MSH2* NM_000251.3; *RAD51D* NM_002878.4.

**Table 4:** Characteristics of patients diagnosed with GC, both carriers and non-carriers, who underwent MGPT.

Variables	All (n = 37)	CARRIERS (n = 5)	NON-CARRIERS (n = 32)	*p*-Value
**Sex**				
Male	14 (100)	3 (21.43)	11 (78.58)	
Female	23 (100)	2 (8.70)	21 (91.30)	0.346
**Median age and IQR (year)**	42 (34–49)	51 (48–59)	42 (34–48)	0.095
**Age at diagnosis**				
<50	28 (100)	2 (7.14)	26 (92.86)	
≥50	9 (100)	3 (33.33)	6 (66.67)	0.081
**Smoking** ^§^				
Ever	15 (100)	1 (6.67)	14 (93.33)	
Never	20 (100)	3 (15.00)	17 (85.00)	0.619
Unknown	2 (100)	1 (50.00)	1 (50.00)	
**Alcohol** ^§^				
Yes	21 (100)	3 (13.64)	19 (86.36)	
No	13 (100)	1 (7.69)	12 (92.31)	1.000
Unknown	2 (50.00)	1 (50.00)	1 (50.00)	
***H. pylori* infection**				
Yes	8 (100)	1 (12.50)	7 (87.50)	
No	7 (100)	0 (0.00)	7 (100.00)	0.802
Unknown	22 (100)	4 (18.18)	18 (81.82)	
**Histology according to Lauren**				
Diffuse	24 (100)	2 (8.33)	22 (91.67)	
Intestinal	3 (100)	2 (66.67)	1 (33.33)	0.075
Mixed	5 (100)	0 (0.00)	5 (100.00)	
Unknown	4 (100)	1 (20.00)	4 (80.00)	
**Anatomic location**				
Cardia	6 (100)	0 (0.00)	6 (100.00)	
Non-Cardia	31 (100)	5 (16.13)	26 (83.87)	0.567
**AJCC Staging at diagnosis**				
Early Stage	18 (100)	3 (16.67)	15 (83.33)	
Late Stage	18 (100)	2 (11.11)	16 (88.89)	
Unknown	1 (100)	0 (0.00)	1 (100.00)	1.000
**Other tumors**				
Yes	8 (100)	3 (37.50)	5 (62.50)	
No	29 (100)	2 (6.90)	27 (93.10)	0.057
**Family History**				
Yes	31 (100)	5 (16.19)	26 (83.87)	
No	5 (100)	0 (0.00)	5 (100.00)	1.000
Unknown	1 (100)	0 (0.00)	1 (100.00)	
GC	18 (100)	5 (27.78)	13 (72.22)	
Non GC	18 (100)	0 (0.00)	18 (100.00)	**0.045**
HBOC-related *	17 (100)	3 (17.65)	14 (82.35)	
Non HBOC-related	19 (100)	2 (10.53)	17 (89.47)	0.650
LS-related *	12 (100)	4 (33.33)	8 (66.67)	
Non LS-related	24 (100)	1 (4.17)	23 (95.3)	**0.036**

HBOC Hereditary Breast and Ovarian Cancer, IQR interquartile range, LS Lynch syndrome, * with the exception of pancreatic cancer. ^§^ *p*-value excluding missing values.

## Data Availability

The data presented in this study are available upon request from the corresponding author. The data are not publicly available due to ethical restrictions.

## Supplementary Materials

The following supporting information can be downloaded at: https://www.mdpi.com/article/10.3390/genes14051077/s1. Table S1: List of genes included in the panel used for the present study, Table S2: Individual clinical features and detailed family history of the 54 GC patients, Table S3: Detailed genetic testing results from both SGT and MGPT.

## Abbreviations

AJCC	American Joint Committee on Cancer
BC	breast cancer
CRC	colorectal adenocarcinoma
DGC	diffuse gastric cancer
FAP	Familial Adenomatous Polyposis
FH	family history
FIGC	Familial Intestinal Gastric Cancer
GAPPS	Gastric Adenocarcinoma and Proximal Polyposis of the Stomach
GC	gastric cancer
GIST	gastrointestinal stromal tumor
*H. pylori*	*Helicobacter pylori*
HBOC	Hereditary Breast and Ovarian Cancer
HDGC	Hereditary Diffuse Gastric Cancer
HR	Homologous Recombination
IARC	International Agency for Research on Cancer
IEO	European Institute of Oncology
IGC	intestinal gastric cancer
IGCLC	International Gastric Cancer Linkage Consortium
IQR	interquartile range
LOVD	Leiden Open Variation Database
LS	Lynch syndrome
MGPT	multigene panel testing
MMR	mismatch repair
MSI	microsatellite instability
PH	personal history
PARPi	poly (ADP-ribose) polymerase inhibitors
PV	pathogenic variant
SGT	single gene testing
VUS	variant of unknown significance
