# Supplementary material for: Hereditary Gastric Cancer: Single-Gene or Multigene Panel Testing? A Mono-Institutional Experience
Source: Genes (Basel). 2023 May 13;14(5):1077. doi: 10.3390/genes14051077 (PMC10218517; doi:10.3390/genes14051077)
Supplement: Supplementary file 1 [file genes-14-01077-s001.zip › Table S1.pdf]

Table S1. List of genes included in the panel used for the present study.

APC	*CDH1*	*MLH1*	*PALB2*	*RAD51C*
ATM	*CDK4*	*MRE11A*	*PIK3CA*	*RAD51D*
BARD1	*CDKN2A*	*MSH2*	*PMS2*	*STK11*
BRCA1	*CHEK2*	*MSH6*	*PMS2CL*	*TP53*
BRCA2	*EPCAM*	*MUTYH*	*PTEN*	*XRCC2*
BRIP1	*FAM175A*	*NBN*	*RAD50*	
