# Supplementary material for: Hereditary Gastric Cancer: Single-Gene or Multigene Panel Testing? A Mono-Institutional Experience
Source: Genes (Basel). 2023 May 13;14(5):1077. doi: 10.3390/genes14051077 (PMC10218517; doi:10.3390/genes14051077)
Supplement: Supplementary file 1 [file genes-14-01077-s001.zip › Table S2.pdf]

Table S2. **Individual clinical features and detailed family history of the 54 GC patients.**

ID	Sex	Age at diagnosis	Lauren Histology	Anatomic Location	AJCC Prognostic Staging	Other tumor (age at diagnosis)	Relatives with GC (Lauren histology, age at diagnosis)	FH of other cancers (age at diagnosis)*
**1**	F	40	Intestinal	Unknown	IVB	NSCLC (47)	FDR (DGC, 39), TDR (NOS, 78)	SDR PC (80)
**2**	F	68	Intestinal	Non-Cardia	I	CRC (52), Other (79)		FDR PC (52) and CRC (60), FDR EC (44) and CRC (46), FDR CRC (54), FDR PrC (65), FDR BC (42), FDR CRC (49), SDR CRC (28)
**3**	F	45	Diffuse	Non-Cardia	I		M.S.: FDR (IGC, 72)	M.S.: SDR PrC (70), SDR liver (58), SDR liver (79); P.S.: SDR OvCa (50), SDR BC (33), SDR bladder (52), SDR HD (48), SDR lung (49)
**4**	M	45	Diffuse	Cardia	IIA		SDR (NOS, 80)	TDR NOS (58)
**5**	F	40	Diffuse	Non-Cardia	I		P.S.: SDR (NOS, 79)	M.S.: TDR BC (75)
**6**	F	69	Diffuse	Non-Cardia	I	DBC (45), CRC (69)	SDR (NOS, 42)	FDR BBC (33,33), FDR PC (67), SDR CRC (79), TDR BC (60), TDR CRC (55), TDR CRC (66)
**7**	F	61	Diffuse	Non-Cardia	III	Bilateral DBC (42,52)		M.S.: FDR EC (72), SDR NOS (72) – P.S.: SDR BBC (59, 67), SDR BC (60)
**8**	M	56	Mixed	Non-Cardia	III			FDR liver (68)
**9**	F	36	Diffuse	Non-Cardia	I		SDR (NOS, 43), TDR (NOS, 76)	SDR BC (45), SDR PrC (81)
**10**	F	42	Diffuse	Cardia	III			FDR kidney (67)
**11**	F	44	Mixed	Non-Cardia	IIB	DBC (34,43,47)	SDR (NOS, 96)	FDR BC (51), SDR BC (53) and lung (61), SDR PrC (80) and CRC (90)
**12**	M	36	Unknown	Cardia	IV	HD (34)		SDR BC (59), SDR osteosarcoma (72)
**13**	F	48	Diffuse	Non-Cardia	I		SDR (NOS, 55)	SDR lung (37), SDR BC (56), SDR lung (70), TDR CRC (60)
**14**	M	60	Intestinal	Non-Cardia	IIA	T.n. (31), PrC (64), SCC (75)		FDR PC (?), FDR PC (?)
**15**	M	50	Mixed	Cardia	III		FDR (NOS, 77), SDR (NOS, 75)	
**16**	F	26	Mixed	Non-Cardia	IV			SDR bladder cancer (84), SDR melanoma (81)
**17**	F	32	Unknown	Non-Cardia	IV			
**18**	M	31	Diffuse	Cardia	III			FDR bladder carcinoma in situ (19), SDR OvCa (75)
**19**	F	34	Diffuse	Non-Cardia	IV			
**20**	F	35	Unknown	Non-Cardia	IV			
**21**	F	54	Mixed	Non-Cardia	I		P.S.: FDR (NOS, 64)	M.S.: SDR CRC (70); P.S.: SDR BC (54)
**22**	M	30	Diffuse	Non-Cardia	0		FDR (NOS, 64), SDR (NOS, 51), TDR (NOS, ?)	
**23**	M	59	Intestinal	Non-Cardia	I	CRC (43), PrC (56)	SDR (NOS, 76)	FDR CRC (50), SDR CRC (45)
**24**	F	48	Mixed	Non-Cardia	IA	DBC (33), TNBC (50)		SDR OvCa (45), SDR OvCa (65), SDR BC (50), TDR BC (31), TDR CRC (41)
**25**	M	34	Diffuse	Non-Cardia	I			SDR PrC (75) and leukemia (75)
**26**	F	49	Diffuse	Non-Cardia	I		FDR (NOS, 40), FDR (NOS, 40)	FDR CRC (52) and urothelial cancer (62), SDR CRC (61)
**27**	M	42	Diffuse	Non-Cardia	IV			SDR sarcoma (32), SDR lung (52)
**28**	M	47	Diffuse	Non-Cardia	I			FDR lung (67), SDR LBC (67), SDR BC (82), TDR BC (38)
**29**	F	52	Diffuse	Cardia	I		FDR (NOS, 53)	
**30**	F	36	Mixed	Non-Cardia	Unknown			
**31**	M	31	Unknown	Cardia	IV			SDR lung (90)

**32**	F	55	Intestinal	Unknown	IIA	Bilateral DBC (39,59)		FDR BBC (53, 60), SDR BC (55), SDR thyroid (50)
**33**	F	42	Diffuse	Non-Cardia	IV			SDR BC (72), SDR GI neoplasia (54)
**34**	F	35	Diffuse	Unknown	Unknown		FDR (DGC, 18)	FDR lymphoma (22)
**35**	F	54	Diffuse	Non-Cardia	IIA	OvCa (55)		FDR haematological neoplasia (70), SDR gynecological neoplasia (36), TDR BC (35)
**36**	M	49	Diffuse	Non-Cardia	IV		M.S.: FDR (NOS, 72); P.S.: SDR (NOS, 62)	M.S.: SDR lymphoma (57), SDR thyroid (80), SDR liver (80). P.S.: FDR lymphoma (49), SDR BC (62)
**37**	F	42	Diffuse	Non-Cardia	I		FDR (NOS, 44); SDR (NOS, 48)	
**38**	M	29	Diffuse	Non-Cardia	I		FDR (DGC, 40), FDR (DGC, 36), FDR (DGC, 16), FDR (DGC, 23)	
**39**	F	23	Diffuse	Non-Cardia	IV			SDR BC (46) and EC (51), SDR CRC (75)
**40**	F	41	Diffuse	Non-Cardia	I	Bilateral LBC (36, 44)	SDR (NOS, 62)	FDR LBC (45), SDR BC (62)
**41**	F	42	Diffuse	Non-Cardia	III		SDR (NOS, ?)	FDR lung (64), SDR PC (?)
**42**	M	51	Unknown	Non-Cardia	I	CRC (66), PC (67)	FDR (NOS, 74)	FDR BC (41), FDR colorectal polyposis
**43**	F	41	Diffuse	Non-Cardia	IV		SDR (NOS, 53)	FDR GIST (59), SDR lung (60)
**44**	F	27	Diffuse	Non-Cardia	I		SDR (DGC, 41)	FDR DBC (54), SDR BC (70)
**45**	F	43	Intestinal	Unknown	Unknown			FDR TNBC (43), FDR BC (53), SDR esophageal neoplasia (?)
**46**	F	35	Diffuse	Non-Cardia	III			NOS (?)
**47**	F	71	Diffuse	Non-Cardia	III		FDR (IGC, 87), SDR (NOS, 55), SDR (NOS, 89)	
**48**	M	62	Intestinal	Unknown	I		FDR/ patient 49 (IGC, 70)	FDR BC (64) and bladder (71) (patient 49), FDR CRC (44), FDR bladder (54), FDR CRC (47), FDR BC (58), FDR CRC (44), SDR BC (35), SDR sarcoma (51), TDR BC (60)
**49§**	F	70	Intestinal	Non-Cardia	III	DBC (64), Urotelial cancer (71)	FDR/patient 48 (IGC, 62)	FDR CRC (44), FDR bladder (54), FDR CRC (47), FDR BC (58), FDR CRC (44), SDR BC (35), SDR sarcoma (51), TDR BC (60)
**50**	F	45	Diffuse	Non-Cardia	III			
**51**	F	45	Diffuse	Non-Cardia	I			SDR CRC (47), SDR lung (56)
**52**	F	36	Mixed	Non-Cardia	IIA			SDR lung (62), SDR NOS (53)
**53**	M	34	Diffuse	Non-Cardia	IV		SDR (NOS, 62),SDR (NOS, 62)	SDR EC (45)
**54**	F	45	Diffuse	Non-Cardia	IV			Unknown

AJCC American Joint Committee on Cancer, BC breast cancer, BBC bilateral breast cancer, CRC colorectal cancer, DBC ductal breast cancer, DGC diffuse gastric cancer, EC endometrial cancer, FH family history, GC gastric cancer, GI gastrointestinal, GIST gastrointestinal stromal tumor, HD Hodgkin disease, H&N head&neck neoplasia, IGC intestinal gastric cancer, LBC lobular breast cancer, NOS not otherwise specified, NSCLC non-small cell lung cancer, OvCa ovarian cancer, PC pancreatic cancer, PrC prostate cancer, SCC squamous cell carcinoma, T.n. testis neoplasia, TNBC triple negative breast

cancer, FDR first-degree relative, SDR second-degree relative, TDR third-degree relative, M.S. maternal side, P.S. paternal side. *If not otherwise specified, FH has been reported for the most relevant side of the family (paternal or maternal) § proband
