# Supplementary material for: Hereditary Gastric Cancer: Single-Gene or Multigene Panel Testing? A Mono-Institutional Experience
Source: Genes (Basel). 2023 May 13;14(5):1077. doi: 10.3390/genes14051077 (PMC10218517; doi:10.3390/genes14051077)
Supplement: Supplementary file 1 [file genes-14-01077-s001.zip › Table S3.pdf]

Table S3. Detailed genetic testing results from both SGT and MGPT.

ID	SGT gene/genes analysed	SGT Type	SGT RESULTS	MGPT	MGPT RESULTS	PV Details	VUS Details
**1**	*CDH1*	UNK	N	no	n.a.		
**2**	*MSH2*	FAM	P	no	n.a.	*MSH2*: c.1216C>T (p.Arg406Ter)	
**3**	*CDH1, MLH1, MSH2, MSH6, PMS2, EPCAM**	UNK	N	yes	U		*RAD50*: c.3036+5G>A (p.?)
**4**	*CDH1*	UNK	N	yes	N		
**5**	*CDH1, MLH1, MSH2, MSH6, PMS2, EPCAM**	UNK	N	yes	U		*BRCA1*: c.547+14del (p.?)
**6**	*BRCA1, BRCA2*	UNK	N	yes	N		
**7**	*BRCA1, BRCA2*	UNK	P	no	n.a.	*BRCA2*: c.8633-1G>A (p.?)	
**8**	*CDH1*	KNOWN**	N	no	n.a.		
**9**	*CDH1, MLH1, MSH2, MSH6, PMS2, EPCAM**	UNK	N	yes	N		
**10**	*CDH1*	UNK	N	yes	N		
**11**	*CDH1, BRCA1, BRCA2*	UNK	N	yes	N		
**12**	*CDH1, MLH1, MSH2, MSH6, PMS2, EPCAM**	UNK	N	yes	N		
**13**	*CDH1*	UNK	N	yes	P	*RAD51D*: c.1A>G (p.Met1Val)	*RAD51D*: c.907A>G (p.Thr303Ala)
**14**	*CDH1, MLH1, MSH2, MSH6, PMS2, EPCAM**	UNK	N	yes	N		
**15**	*CDH1, MLH1, PMS2*	UNK	N	yes	N		
**16**	*CDH1*	UNK	N	yes	U		*BRCA2*: c.5492T>C (p.Ile1831Thr)
**17**	*CDH1*	UNK	N	yes	N		
**18**	*CDH1*	UNK	N	yes	N		
**19**	*CDH1*	UNK	N	yes	N		
**20**	*CDH1*	UNK	N	yes	N		
**21**	*CDH1*	UNK	N	no	n.a.		
**22**	CDH1	UNK	N	yes	N		
**23**	*MSH2, MSH6, EPCAM**	UNK	P	yes	P	*MSH2*: c.339_340del (p.Asn115Ter)	
**24**	*BRCA1, BRCA2*	UNK	P	no	n.a.	*BRCA1*: c.5278-2A>T (p.?)	
**25**	*CDH1*	UNK	N	yes	U		*RAD50*: c.980G>A (p.Arg327His)
**26**	*CDH1*	UNK	N	yes	U		*ATM*: c.1898+3A>G (p.?)<br>*APC*: c.6019T>C (p.Tyr2007His)<br>*MUTYH*: c.925C>T (p.Arg309Cys)<br>*MUTYH*: c.1276C>T (p.Arg426Cys)
**27**	*CDH1*	UNK	N	yes	U		*STK11*: c.1211C>T (p.Ser404Phe)
**28**	*CDH1*	UNK	N	yes	N		
**29**	*CDH1*	UNK	N	yes	U		*APC*: c.2584A>G (p.Asn862Asp)<br>*MUTYH*: c.1276C>T (p.Arg426Cys)

**30**	*MSH2, MSH6, EPCAM**	UNK	N	yes	N		
**31**	*CDH1*	UNK	N	yes	U		*RAD50*: c.3480T>G (p.Ile1160Met)<br>*BARD1*: c.2251C>T (p.Arg751Trp)
**32**	*BRCA1, BRCA2*	UNK	N	no	n.a.		
**33**	*CDH1*	UNK	N	yes	U		*PALB2*: c.1784A>C (p.Asp595Ala)
**34**	*CDH1*	UNK	N	no	n.a.		
**35**	*CDH1, BRCA1,* BRCA2	UNK	U	yes	U		*BRCA2*: c.796T>G (p.Phe266Val)<br>*BRCA2*: c.475+11A>C (p.?)<br>*ATM*: c.1790C>T (p.Pro597Leu)
**36**	*CDH1*	UNK	N	yes	U		*MUTYH*: c.667A>G (p.Ile223Val)
**37**	*CDH1*	UNK	N	no	n.a.		
**38**	*CDH1*	UNK	P	no	n.a.	*CDH1*: c.1792C>T (p.Arg598Ter)	
**39**	*CDH1, MLH1, MSH2, MSH6, PMS2, EPCAM**	UNK	N	yes	N		
**40**	*CDH1, BRCA1, BRCA2*	UNK	P	no	n.a.	*CDH1*: c.833-476_1138-464del (p.Gly278ValfsTer7)	
**41**	*CDH1*	UNK	N	no	n.a.		
**42**	*MLH1, MSH2, MSH6, PMS2, EPCAM**	UNK	N	yes	P	*ATM*: c.2413C>T (p.Arg805Ter)	
**43**	*CDH1*	UNK	N	no	n.a.		
**44**	*CDH1*	UNK	N	yes	U		*MUTYH*: c.56G>A (p.Arg19Gln)
**45**	*BRCA1*	FAM	N	no	n.a.		
**46**	*CDH1*	UNK	N	no	n.a.		
**47**	*CDH1*	UNK	N	yes	N		
**48**	*BRCA2*	FAM	P	no	n.a.	*BRCA2*: c.67+1G>A (p.?)	
**49**	*CDH1, BRCA1, BRCA2, MLH1, MSH2, MSH6, PMS2, EPCAM**	UNK	P	yes	P	*BRCA2*: c.67+1G>A (p.?)	*ATM*: c.5890A>G (p.Lys1964Glu)
**50**	*CDH1*	UNK	N	yes	N		
**51**	*CDH1*	UNK	N	no	n.a.		
**52**	*CDH1*	UNK	N	yes	U		*MLH1*: c.283T>G (p.Ser95Ala)<br>*NBN*: c.1079C>A (p.Thr360Asn)
**53**	*CDH1*	UNK	P	yes	P	*CDH1*: c.2416G>T (p.Glu806Ter)	
**54**	*CDH1, BRCA1, BRCA2, MLH1, MSH2, MSH6, PMS2, EPCAM**	UNK	N	yes	N		

SGT Single-Gene Testing, MGPT Multigene Panel Testing, PV Pathogenic Variant (including C4 and C5), VUS Variant of Unknown Significance, HDGC Hereditary Diffuse Gastric Cancer, FIGC Familial Intestinal Gastric Cancer, HBOC Hereditary Breast Ovarian Cancer, UNK search of unknown mutations, FAM search for the familial mutation, P positive, N normal, U uncertain, * search only for large rearrangements, ** search for the mutation previously identified in the tumor sample, n.a. not applicable

Reference sequences (Human Feb. 2009 - GRCh37/hg19 Assembly): *APC* NM_000038.6; *ATM* NM_000051.4; *BARD1* NM_000465.4; *BRCA1* NM_007294.4; *BRCA2* NM_000059.4; *CDH1* NM_004360.5; *MLH1* NM_000249.4; *MSH2* NM_000251.3; *MUTYH* NM_001128425.2; *NBN* NM_002485.5; *PALB2* NM_024675.4; *RAD50* NM_005732.4; *RAD51D* NM_002878.4; *STK11* NM_000455.5
